# Delayed presentation of traumatic diaphragmatic hernia complicated by bowel obstruction and perforation: a case report

**DOI:** 10.1097/MS9.0000000000001091

**Published:** 2023-07-24

**Authors:** Krishna Kumar Yadav, Ranjeet Ghimire, Ranjit Rauniyar, Rebanta Khadka, Arun Batsa Lamsal, Laxman Khadka, Rupesh Kumar Yadav, Pratibha Yadav

**Affiliations:** a Department of General Surgery, Tribhuvan University Teaching Hospital, Maharajgunj; b Institute of Medicine, Tribhuvan University, Maharajgunj Medical Campus; c Nepalese Army Institute of Health Science; d Kathmandu Medical College, Kathmandu, Nepal

**Keywords:** bowel obstruction, bowel perforation, post-traumatic diaphragmatic hernia

## Abstract

Post-traumatic diaphragmatic hernia is a rare but life-threatening condition resulting from a traumatic injury to the diaphragm. We present a case of a 48-year-old man with a history of fall injury and a delayed presentation of a right-sided diaphragmatic hernia with subsequent bowel obstruction and perforation in a patient with a history of trauma and tuberculosis. The patient underwent exploratory laparotomy with manual reduction of the herniated bowel, primary repair of the diaphragmatic defect, and the creation of a double-barrel ileostomy. This case highlights the importance of considering traumatic diaphragmatic hernia in patients with a history of trauma presenting with abdominal symptoms. Timely diagnosis and surgical intervention are crucial in preventing serious complications associated with this condition.

## Introduction

Highlights

- Rare and life-threatening case of delayed traumatic diaphragmatic hernia complicated by bowel obstruction and perforation.
- A 48-year-old man with a prior history of fall injury and tuberculosis experienced a delayed presentation of a right-sided diaphragmatic hernia.
- Computed tomography imaging confirmed the diaphragmatic hernia and guided the surgical intervention.
- Traumatic diaphragmatic hernia should be considered in trauma patients, even with other comorbidities.
- Early recognition, accurate imaging, and prompt surgical management are crucial for improved outcomes.

Diaphragmatic hernia is characterized by a defect in the diaphragm that allows the abdominal contents to herniate into the thoracic cavity. Traumatic diaphragmatic hernias (TDHs) can result from both blunt and penetrating trauma and pose a diagnostic and therapeutic challenge^1^. Delayed presentations of TDHs are uncommon but can lead to serious complications such as bowel obstruction and perforation^2^. The factors that contribute to the delay in diagnosing diaphragmatic rupture after trauma include a lack of immediate symptoms, the oversight of the diaphragmatic injury during the initial assessment, or the misinterpretation of radiological findings^3^.

## Case report

A 48-year-old man presented with a complaint of abdominal pain for 10 days with multiple episodes of vomiting with a history of not being able to pass stool and flatus for 8 days. He had a history of pulmonary tuberculosis 14 years back and a history of fall injuries 6 months back.

On examination, the abdomen was distended, and guarding and rigidity were present, along with loss of liver dullness and an absent bowel sound with decreased air entry on the bilateral chest on the basal area.

Hematological and biochemical parameters showed a slightly raised total leukocyte count of 11 300 with 85% neutrophils and 11% lymphocytes; however, other parameters were normal. Urine and creatinine levels were within the normal range, as were the levels of sodium, potassium, liver enzymes, amylase, lipase, total protein, and albumin.

**Figure 1:**
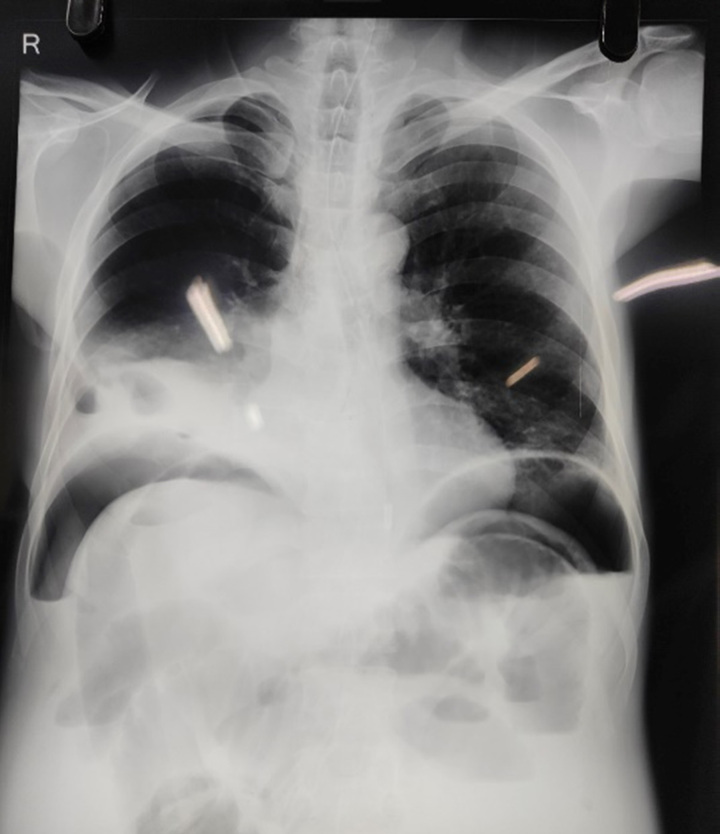
Preoperative chest radiograph showing radio-opaque area over the lower one-third of right lung suggestive of bowel herniation.

**Figure 2:**
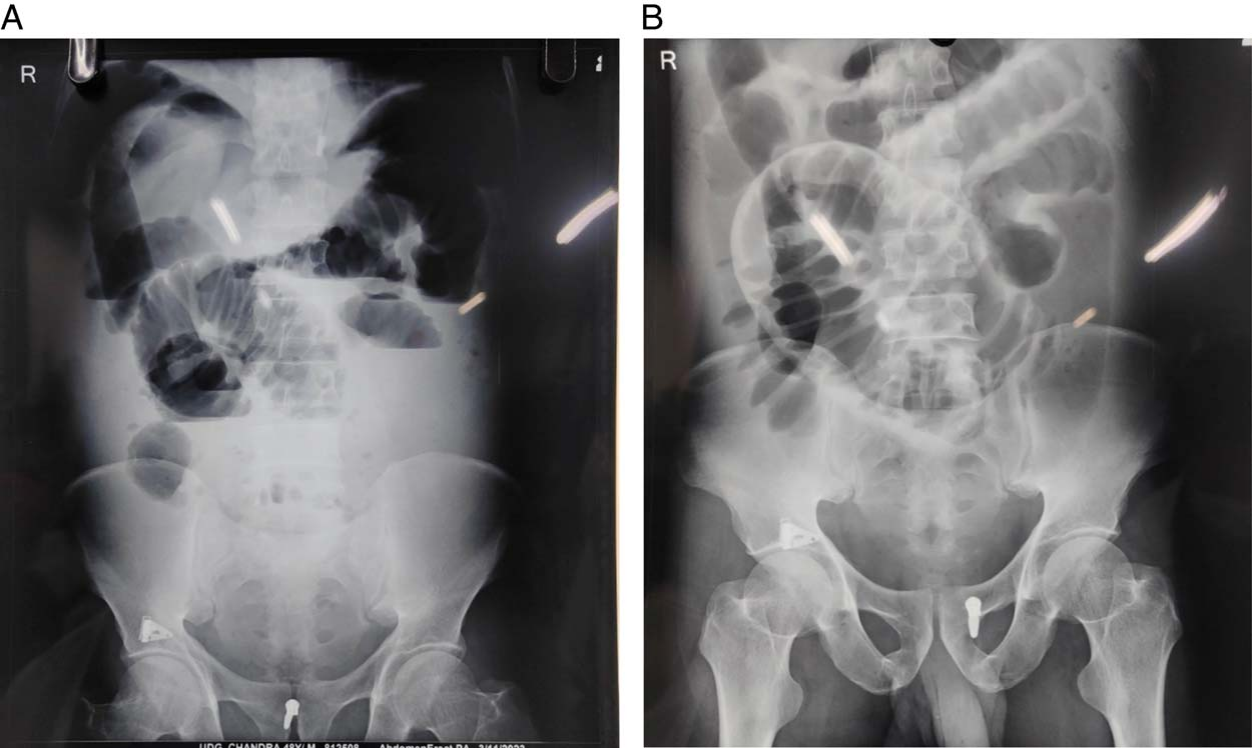
(A) Erect radiograph abdomen showing air-fluid level suggesting small bowel obstruction. (B) Supine radiograph abdomen showing distended small bowel loops involving whole small bowel.

The preoperative chest radiograph revealed a suspicious opacity in the right lower zone, shown in Figure 1A, whereas the abdominal radiograph showed distended small bowel loops, as shown in Figure 2A, B. A contrast-enhanced computed tomography (CECT) scan was subsequently performed for further evaluation and confirmation of a diaphragmatic hernia with bowel obstruction.

**Figure 3:**
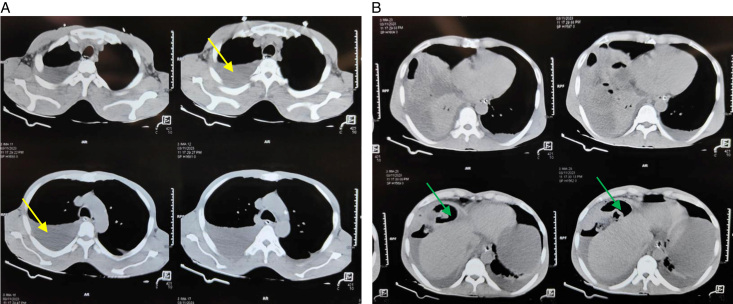
(A) Contrast-enhanced computed tomography (CECT) thorax showing collapsed right lung (yellow arrow). (B) CECT abdomen showing herniated small bowel loops over the liver (green arrow).

On CECT abdomen and pelvis, a defect of size 3 cm was noted in the right hemidiaphragm, through which there was herniation of distal ileal loop and mesentery shown in Figure 3A, B, causing closed-loop obstruction of the herniated bowel with thickening of the wall and mural stratification. Dilatation of the jejunal and ileal loops proximal to the herniation was observed, along with moderate fluid in the abdomen and pelvis and moderate right and mild left pleural effusions.

**Figure 4:**
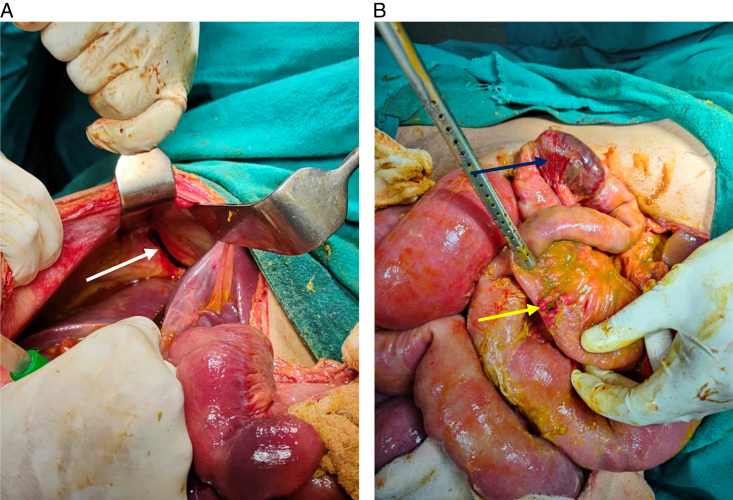
(A) Intraoperative view showing a right-sided diaphragmatic defect of 3×1 cm. (B) 2×2 cm perforation at the ileum (yellow arrow), 30 cm from the ileocolic junction and gangrenous ileum of 5 cm, which is 20 cm from the ileocolic valve (blue arrow).

The patient was admitted with a diagnosis of right-sided diaphragmatic hernia with herniating closed-loop ileal obstruction and ileal perforation. On exploratory laparotomy, about 1400 ml of straw-colored fluid was found in the peritoneum. There was a closed-loop ileal obstruction with a perforation of 2×2 cm, 30 cm from the ileocolic junction, and a gangrenous ileum of 5 cm, which is 20 cm from the ileocolic valves shown in Figure 4B. The diaphragmatic defect in the right dome of the diaphragm was 3×1 cm, as shown in Figure 4A.

The patient underwent manual reduction of the ileal loop, followed by a thorough wash of the right pleural cavity, and a chest tube was placed. The right-sided diaphragmatic defect was primarily repaired. The gangrenous ileal segment was resected out, followed by double-barrel ileostomy.

The patient had a successful recovery without complications and was discharged after 28 days. A follow-up after 2 months confirmed their well-being and the absence of any issues.

The plan is for ileostomy reversal in 3 months, and the patient is in continuous follow-up.

## Discussion

Post-traumatic diaphragmatic hernia is defined as the displacement of intra-abdominal organs into the chest through a pathological orifice in the diaphragm resulting from trauma^4^. While its immediate presentation following trauma is well-documented, cases of delayed presentation with complications such as bowel obstruction and perforation are particularly rare and clinically challenging.

The incidence of TDH is estimated to be 0.8–5% among all thoracoabdominal injuries^5,6^. However, the incidence of delayed presentations of TDH complicated by bowel obstruction and perforation within this subset is even lower, ranging from 0.17 to 6%^7^. In a retrospective study conducted by Smith *et al*.^8^, out of 276 cases of TDH, only 8% presented with delayed symptoms, and among them, only a small proportion developed complications such as bowel obstruction and perforation. Blunt trauma, including falls, motor vehicle accidents, or direct blows to the chest and abdomen, is the main cause of TDH (81%)^9^. The left hemidiaphragm is the most frequently affected site, accounting for 80–90% of cases^5,6^.

Diagnosis of TDH can be challenging due to its subtle or nonspecific symptoms, which can be masked by other underlying conditions. However, symptoms such as shoulder and/or epigastric pain, respiratory distress, and intrathoracic bowel sounds can indicate diaphragmatic defects. In cases where the defect is detected later on and becomes chronic, patients may present with symptoms suggestive of partial or complete intestinal obstruction^10^.

In this case, the patient had a prior history of trauma 6 months prior to presentation, raising suspicion for the delayed manifestation of TDH. While the presence of a past history of tuberculosis added complexity to the differential diagnosis, as it could have potentially led to intestinal obstruction. However, the subsequent development of intestinal obstruction symptoms and radiological findings confirmed the diagnosis of a rare right-sided TDH.

Radiological diagnosis is the mainstay for the diagnosis of diaphragmatic hernias^11^, which can be asymptomatic and unnoticed sometimes^10^. An initial radiograph of the chest and abdomen can be performed to assess for any signs, such as an elevated hemidiaphragm, bowel gas in the chest cavity, or abnormal bowel gas patterns, which can raise suspicion of a diaphragmatic hernia^11^. Computed tomography (CT) is the preferred imaging modality for diagnosing diaphragmatic injuries in a clinically or radiologically suspected patient^12^, offering valuable information for surgical planning.

TDHs are classified into three types based on the time interval for diagnosis^13^:

Type 1 hernia – when the diagnosis is made immediately following trauma.

Type 2 hernia – when the diagnosis is made within the recovery period.

Type 3 hernia – when the diagnosis is made when the patient presents with ischemia or perforation of herniated organs.

Our case falls into the Type 3 category, as the patient only presented after ischemia and perforation of a herniated closed ileal loop.

The surgical management of TDHs involves the reduction of herniated organs, repair of the diaphragmatic defect, and appropriate drainage^14^. In our case, CT findings confirmed the presence of a diaphragmatic hernia and guided the subsequent surgical intervention, which involved exploratory laparotomy with manual reduction of the ileal loop. Primary repair of the right-sided diaphragmatic defect was performed, and a double-barrel ileostomy was created. This comprehensive surgical approach aimed to relieve bowel obstruction, restore normal anatomy, and prevent further complications.

This case report highlights several noteworthy aspects. Firstly, the delayed presentation of TDH complicated by bowel obstruction and perforation is an uncommon and challenging scenario. The presence of a prior history of trauma, despite a significant time lapse, underscores the significance of considering TDH in the differential diagnosis. Secondly, the radiological findings, both the initial radiograph and subsequent CT scan, played a pivotal role in confirming the diagnosis and guiding the surgical intervention. These imaging modalities can aid in detecting diaphragmatic, even in cases where symptoms may be absent or overlooked.

Timely recognition and effective management of TDHs play a vital role in preventing complications and improving patient outcomes. The rarity of this case underscores the importance of increased clinical awareness and multidisciplinary collaboration involving radiologists, surgeons, and critical care physicians. Furthermore, this case report contributes to the existing literature on TDHs, particularly the delayed presentation with bowel obstruction and perforation. Given the rarity and intricacy of such cases, there is a need for further documentation and research to expand our understanding and enhance clinical approaches.

## Conclusion

Delayed TDHs are rare but potentially life-threatening conditions that require prompt recognition and intervention. This case emphasizes the importance of considering diaphragmatic hernias in patients with a history of trauma, even if there is a delay in presentation. CT plays a vital role in diagnosing diaphragmatic injuries, and surgical intervention, including reduction of herniated organs and repair of the diaphragmatic defect, remains the mainstay of treatment. Early diagnosis and timely surgical intervention are essential to prevent serious complications associated with TDHs.

## Ethical approval

Ethical approval was not necessary for this case report as it involves the presentation of a single patient’s medical history and treatment. The case report does not involve experimental interventions or research on human subjects. It focuses on the clinical management and challenges associated with post-traumatic diaphragmatic hernia. Therefore, ethical approval for this particular case report was not required.

## Consent

Written informed consent was obtained from the parents for the publication of this case report and accompanying images. A copy of the written consent is available for review by the Editor-in-Chief of this journal on request.

## Sources of funding

No funding was received for the study.

## Author contribution

K.K.Y.: had taken the history and was involved in the active management of the case; R.G., R.R., R.K., A.B.L., L.K., and R.K.Y.: were involved in the writing of the manuscript; K.K.Y. and P.Y.: edited and revised the manuscript. All authors have read and approved the final version of the manuscript.

## Conflicts of interest disclosure

The authors declare no conflicts of interest.

## Research registration unique identifying number (UIN)

1. Name of the registry: none.
2. Unique identifying number or registration ID: none.
3. Hyperlink to your specific registration (must be publicly accessible and will be checked): none.

## Guarantor

Krishna Kumar Yadav, Department of General Surgery, Tribhuvan University, Institute of Medicine, Maharajgunj, Kathmandu, Nepal, E-mail: krishna321ydv@gmail.com

## Data availability statement

All available data are within the manuscript itself.

## Provenance and peer review

Not commissioned, externally peer-reviewed.
